# Association between Serum Folate and Insulin Resistance among U.S. Nondiabetic Adults

**DOI:** 10.1038/s41598-017-09522-5

**Published:** 2017-08-23

**Authors:** Jinchao Li, Charlene E. Goh, Ryan T. Demmer, Brian W. Whitcomb, Peng Du, Zhenhua Liu

**Affiliations:** 1 Department of Nutrition, School of Public Health and Health Sciences, University of Massachusetts, Amherst, MA USA; 2 Department of Epidemiology, Mailman School of Public Health, Columbia University, New York, New York, USA; 3 Department of Biostatistics and Epidemiology, University of Massachusetts, Amherst, MA USA; 4 Department of Mathematics and Statistics, University of Massachusetts, Amherst, MA USA; 5 Jean Mayer USDA Human Nutrition Research Center on Aging, Tufts University, Boston, MA USA

## Abstract

Recent studies have suggested that epigenetic alterations, particularly DNA methylation, play a crucial role in the pathogenesis of insulin resistance. Folate is a key source of the one-carbon group for DNA methylation, whereas the association and mechanistic linkage between folate status and insulin resistance remains unclear with very limited experimental support. We performed a cross-sectional study of 1530 nondiabetic adults in the 2011–2012 National Health and Nutrition Examination Survey (NHANES). We examined associations between serum folate and insulin resistance using multiple linear regression models adjusted for potential confounders. We detected a significant inverse relationship for serum folate, where a 25% increase in serum folate was associated with a 3.06% (95% CI, −4.72, −1.37) and 2.77% (95% CI, −4.36, −1.77) decline in HOMA-IR and insulin respectively, and a 2.55% (95% CI, 0.93, 4.21) increase in G/I ratio. Our findings demonstrate that serum folate was inversely associated with insulin resistance in U.S. nondiabetic adults.

## Introduction

Insulin resistance (IR) is a major metabolic abnormality and it raises the risk of developing many disorders, such as type 2 diabetes, obesity, hypertension, cardiovascular disease, and metabolic syndrome^1, 2^. Obesity is the most common cause of insulin resistance, and the global epidemic of obesity has become the central driving force behind increases in insulin resistance and its complications. The etiology of insulin resistance has been subject to intense investigation, and it is now thought that systemic inflammation, activation of unfolded protein response, and ectopic lipid accumulation are important causes of obesity-induced insulin resistance^3^. Recent studies have suggested that epigenetic alterations, particularly DNA methylation, may play a crucial role in the pathogenesis of insulin resistance^4–8^.

Folate, a water-soluble B-vitamin also known as vitamin B9, is a key source of the one-carbon group used in methylation reactions and DNA/RNA synthesis. Low folate status has been associated with an increased risk of neural tube defects, cancer, and coronary heart disease^9^, but the data pertaining the association between folate status and insulin resistance are limited. In this study, we examined the relationship between serum folate and insulin resistance using a large representative sample of nondiabetic U.S. adults.

## Methods

The National Health and Nutrition Examination Survey﻿ (NHA﻿NES) is a cross sectional study designed to collect health and nutritional data from a nationally representative sample of the resident civilian noninstitutionalized U.S. general population. NHANES, conducted by the Centers for Disease Control and Prevention’s (CDC) National Center for Health Statistics (NCHS), employs a multistage stratified probability sample based on selected counties, blocks, households, and persons within households. Subjects participated in a household interview and physical measurements and biochemical measurements from blood and urine were conducted at mobile exam centers (MECs). Study protocols were approved by the NCHS Research Ethics Review Board (Protocol #2011–17), and written informed consent was obtained from all participants. All methods were performed in compliance with the Declaration of Helsinki. Details on the design and methodology of the Continuous NHANES have been previously published^10^.

Of the 5560 adult participants aged 20 years and older in cycles 2011–2012, 2295 subjects were included in fasting glucose and oral glucose tolerance test (OGTT) subsample. 640 participants who had diabetes or took antidiabetic medications were excluded. Diabetes was defined as a self-reported diagnosis of diabetes, or fasting plasma glucose >= 126 mg/dl, or OGTT > = 200 mg/dl. Participants who were taking medications that would interfere with glucose metabolism (steroids or androgens) (n = 23), who were pregnant (n = 1), or who were missing important covariates such as BMI (n = 14), serum folate (n = 33), and serum insulin (n = 54) were excluded. A total of 1530 participants were included in final regression analyses.

### Measurement of serum folate

Serum folate concentrations were measured by isotope-dilution high performance liquid chromatography coupled to tandem mass spectrometry (LC-MS/MS). The assay is performed by combining specimen (275 μL serum or whole blood hemolysate) with ammonium formate buffer and an internal standard mixture. Detailed instructions on specimen collection and processing can be found on the NHANES website^11^.

### Measurement of insulin resistance

Fasting plasma glucose, OGTT and serum insulin were measured at a morning examination session. Participants fasting <9 hours, taking insulin or oral medications for diabetes, or refusing phlebotomy were excluded. Homeostatic model assessment of insulin resistance (HOMA-IR)^12^ and insulin^13^ were used as a measure of insulin resistance, with higher levels representing greater degrees of insulin resistance. We calculated HOMA-IR by multiplying fasting glucose (mmol/L) by fasting insulin (U/mL) and dividing by 22.5^12^. G/I ratio was used as a measure of insulin sensitivity, with higher levels representing greater insulin sensitivity. The G/I ratio was defined as follows: fasting glucose (mg/dl)/insulin (uU/mL)^14^.

### Covariates

Potential confounders were age, gender, race/ethnicity, education, BMI, smoking (none, light, heavy), alcohol consumption (none, moderate, heavy), physical activity (none, moderate, vigorous), supplement intake, total cholesterol, blood pressure, white blood cell count, vitamin B12. The intensity of the physical activities was classified according to tertiles of metabolic equivalents task (MET) scores based on the intensity and frequency of a number of physical activities reported during in the preceding 30 days. Dietary supplement and alcohol consumption data were collected using 24-hour recall. Moderate alcohol consumption was defined as more than none but no more than one drink a day for women and up to two drinks a day for men, according to current guidelines of the USDA^15^. Serum cotinine concentrations in the laboratory dataset were used to classify smoking status: subjects were defined as nonsmokers if concentrations were < 0.016 ng/mL, light smokers or heavy-passive smokers if concentrations were 0.016–0.1ng/mL, and heavy smokers if serum cotinine concentrations were >= 0.1ng/ml. Serum vitamin B12 was measured using the fully automated Roche electrochemiluminescence immunoassay on the Elecsys 170.

### Statistical analyses

Strata and cluster variables provided by the CDC within the dataset were incorporated into the analysis to account for the complex survey design and to obtain proper estimates and SEs; OGTT sample weights were used when analyzing fasting glucose, OGTT, and insulin levels to account for unequal probabilities of selection, noncoverage, and nonresponse. PROC SURVEYMEANS, SURVEYFREQ, and SURVEYREG were employed to calculate geometric means, proportions, and to perform multiple linear regressions. Taylor series linearization was used for variance estimation. We used three models with progressive degrees of adjustment to address confounding. Model I was adjusted for sex, age, race/ethnicity, education, BMI. Model II was further adjusted for smoking, alcohol consumption, use of supplements, and physical activity. Model III was further adjusted for total cholesterol, blood pressure, white blood cell count, vitamin B12. Serum folate, HOMA-IR, Insulin, GI ratio, WBC, Vitamin B12 were log transformed to account for skewed distribution, with results presented after retransformation to the original scale. In addition, continuous measures of serum folate levels were categorized into quartiles and used in regression models to allow for non-linearity. Tests of linear trend were performed using quartiles of serum folate levels as a continuous variable. We also examined effect modification by performing stratified analysis by categories of gender, alcohol consumption, and use of supplements. *p* values < 0.05 were considered significant. All statistical analyses were conducted with SAS 9.4 software (SAS Institute).

## Results

The demographic characteristics of the sample population are presented in Table 1. The study sample consisted of 785 men and 745 women. In this study population, males and older adults had a higher value of HOMA-IR, although this difference was not statistically significant. HOMA-IR varied significantly by race/ethnicity (*p* = 0.005); and was higher for the Mexican American populations (2.64), compared with the non-Hispanic white (2.32), non-Hispanic black population (2.35) and other race population (1.94). HOMA-IR was significantly lower in participants who had college education and beyond, in subjects who were underweight, in those who reported taking dietary supplements in the past 24 h, in participants who had high levels of physical activity, and in those who were heavy drinkers. HOMA-IR was not related to smoking status.

**Table 1:** Characteristics of study population, NHANES 2011–2012.

Characteristic	N	Unweighted,%	Weighted,%	HOMA-IR, weighted mean (SE)^1^	*p* Value
Gender					0.111
Female	745	48.69	52.08	2.25 (0.07)	
Male	785	51.31	47.92	2.42 (0.06)	
Age, y					0.433
20–39	635	42.68	41.2	2.22 (0.09)	
40–59	521	34.05	38.28	2.38 (0.12)	
60+	356	23.27	20.53	2.45 (0.11)	
Race/ethnicity					0.005
Non-Hispanic White	618	40.39	68.62	2.32 (0.06)	
Mexican American	164	10.72	7.58	2.64 (0.15)	
Non-Hispanic Black	320	20.92	10.5	2.35 (0.08)	
Other Hispanic	160	10.46	6.13	2.46 (0.11)	
Other Race	268	17.52	7.17	1.94 (0.11)	
Education					0.031
Less than high school	315	20.59	15.62	2.33 (0.16)	
High school	304	19.87	18.01	2.58 (0.12)	
Some college	468	30.59	33.72	2.47 (0.14)	
College or above	443	28.95	32.65	2.07 (0.09)	
BMI					<0.001
Normal weight	508	33.2	32.67	1.44 (0.05)	
Underweight	27	1.76	1.16	1.15 (0.08)	
Overweight	516	33.73	34.69	2.37 (0.09)	
Obese	479	31.31	31.47	3.85 (0.12)	
Alcohol^2^					0.003
None	1096	74.86	71.34	2.49 (0.07)	
Moderate	137	9.36	9.53	2.13 (0.17)	
Heavy	231	15.78	19.13	1.96 (0.09)	
Physical activity					<0.001
Low	504	33.03	30.14	2.72 (0.11)	
Moderate	513	33.62	35.08	2.28 (0.07)	
High	509	33.36	34.77	2.08 (0.07)	
Dietary supplement					0.029
Yes	534	36.55	39.24	2.18 (0.06)	
No	927	63.45	60.76	2.46 (0.08)	
Smoking status					0.994
Nonsmoker	420	27.49	34.47	2.33 (0.08)	
Light smoker	530	34.69	30.18	2.34 (0.11)	
Heavy smoker	578	37.83	35.35	2.33 (0.11)	

^1^Geometric Mean.

^2^Moderate alcohol consumption was defined as more than none but no more than one drink a day for women and up to two drinks a day for men, according to current guidelines of the USDA, 1 drink = 14 g ethanol.

The results of the multiple linear regression analysis between serum folate and insulin resistance are displayed in Table 2. The adjusted analyses revealed a significant inverse association (*p* < 0.01) between serum folate and insulin resistance in model I, II, and III. In model III, a 25% increase in serum folate increase in serum folate was associated with a 3.06% (95% CI, −4.72, −1.37) and 2.77% (95% CI, −4.36, −1.77) decline in HOMA-IR and insulin respectively, and a 2.55% (95% CI, 0.93, 4.21) increase in G/I ratio.

**Table 2:** Percentage Change (95%CI) in Insulin Resistance/Sensitivity Associated with a 25% Increase in Serum Folate Concentrations among U.S. Nondiabetic Adults, NHANES 2011–2012

	Model I^1^		Model II^2^		Model III^3^	
	% (95%CI)	*p*	% (95% CI)	*p*	% (95% CI)	*p*
HOMA-IR^4^	−3.44 (−4.78, −2.08)	<0.001	−3.27 (−4.89, −1.86)	<0.001	−3.06 (−4.72, −1.37)	0.002
Insulin^4^	−3.08 (−4.32, −1.82)	<0.001	−3.00 (−4.32, −1.65)	<0.001	−2.77 (−4.36, −1.77)	0.002
G/I ratio^4^	2.79 (1.54, 4.06)	<0.001	2.79 (1.42, 4.18)	<0.001	2.55 (0.93, 4.21)	0.004

^1^Model I: Adjusted for age, gender (M/F), race/ethnicity (non-Hispanic White, Mexican American, non-Hispanic Black, other Hispanic, other Race), education (less than high school, high school, some college, college or above), BMI (normal weight, underweight, overweight, obese).

^2^Model II: Adjusted for confounding factors in Model I plus smoking (nonsmoker, light smoker, heavy smoker), alcohol consumption (none, moderate, heavy), use of supplements (Y/N), and physical activity (low, moderate, high).

^3^Model III: Adjusted for confounding factors in Model II plus total cholesterol, blood pressure, white blood cell count, vitamin B12.

^4^Results were retransformed back to their original scale.

These associations observed in the overall population persisted after stratifying analyses by gender, race/ethnicity, and drinking, and stayed largely consistent across strata of gender, race/ethnicity, and drinking. When stratified by race/ethnicity, the interactions between race/ethnicity and serum folate were not statistically significant, however, the association between insulin resistance and folate tended to be weaker among other Hispanic and Non-Hispanic Black (Table 3).

**Table 3:** Percentage Change (95%CI) in Insulin Resistance/Sensitivity Associated with a 25% Increase in Serum Folate Concentrations among U.S. Nondiabetic Adults, stratified by gender, race/ethnicity, and alcohol consumption, NHANES 2011–2012.

	HOMA-IR		Insulin		G/I ratio	
	% (95% CI)	*p* ^2^	% (95% CI)	*p* ^2^	% (95%CI)	*p* ^2^
Gender^1^		0.81		0.722		0.622
Male (n = 785)	−3.33 (−5.89, −0.70)		−3.01 (−5.29, −0.69)		2.78 (0.65, 4.95)	
Female (n = 745)	−3.05 (−5.29, −0.75)		−2.79 (−4.98, −0.56)		2.61 (0.31, 4.96)	
Race/ethnicity^1^		0.255		0.168		0.095
Mexican American (n = 164)	−3.76 (−7.45, 0.09)		−3.31 (−6.79, 0.29)		2.95 (−0.64, 6.67)	
Other Hispanic (n = 160)	−0.24 (−5.57, 5.39)		−0.18 (−4.70, 4.56)		0.12 (−3.67, 4.05)	
Non-Hispanic White (n = 618)	−3.30 (−5.30, −1.25)		−2.93 (−4.84, −0.99)		2.64 (0.67, 4.64)	
Non-Hispanic Black (n = 320)	−0.31 (−3.05, 2.50)		−0.17 (−2.96, 2.71)		0.02 (−2.96, 3.10)	
Other Race (n = 268)	−2.77 (−6.67, 1.29)		−2.54 (−6.28, 1.35)		2.36 (−1.49, 6.37)	
Alcohol^1^		0.967		0.864		0.735
None (n = 1096)	−2.94 (−5.30, −0.52)		−2.74 (−4.95, −0.49)		2.62 (0.44, 4.84)	
Moderate (n = 137)	−3.97 (−6.71, −1.15)		−3.88 (−6.43, −1.26)		3.94 (1.13, 6.82)	
Heavy (n = 231)	−3.44 (−6.68, −0.09)		−2.72 (−5.79, 0.44)		2.04 (−1.15, 5.33)	

^1^based on Model III.

^2^ *p* value of Interaction between serum folate and variables.

**Figure 1:**
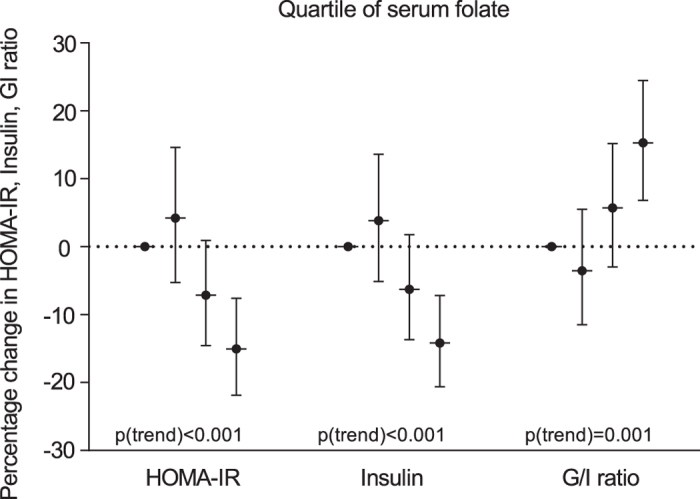
Adjusted percentage change in insulin resistance/sensitivity in relation to quartile of serum folate concentrations. Values are weighted for complex survey design and adjusted for sex, age, race/ethnicity, education, BMI, smoking, alcohol consumption, use of supplements, physical activity, total cholesterol, blood pressure, white blood cell count, and vitamin B12.

In our secondary analyses, we investigated the relationships between quartile of serum folate and measures of insulin resistance (Fig. 1). We observed a statistically significant inverse trend between quartiles of serum folate and HOMA-IR (*p* for trend < 0.001), insulin (*p* for trend < 0.001), and a statistically significant positive trend of increasing G/I ratio (*p* for trend = 0.001) with increasing quartiles of serum folate.

## Discussion

DNA methylation have recently been reported to play a crucial role in the pathogenesis of insulin resistance^4–8^, whereas, whether folate, a key component involved in the DNA methylation reactions, is associated with insulin resistance remains unclear with limited experimental support^16–18^. This study, based on a large nationally representative sample of noninstitutionalized civilian nondiabetic adults in the U.S, demonstrated a strong inverse association between serum folate and insulin resistance. The observed association was independent of age, gender, BMI, and other potential confounding factors, such as supplement intake, alcohol consumption, smoking, physical activity, total cholesterol, race/ethnicity, education, *etc*.

Folate is one of the most important components in one-carbon metabolism network, which is composed of the conversion of homocysteine to methionine, biological methylation and nucleotide (purine and thymidylate) syntheses^19, 20^. When folate is consumed in normal dietary conditions, absorbed folate is metabolized to 5-methyltetrahydrofolate (5-methylTHF). 5-methylTHF supplies a methyl group to convert homocysteine to methionine via the vitamin B12-dependent methionine synthase reaction. Methionine is then converted to S-adenosylmethionine (SAM), a universal methyl donor for numerous methylation reactions including the methylation of DNA, RNA^21^. Folate deficiency can cause hyperhomocysteinemia^22^, which is a powerful risk factor for vascular damage and cardiovascular disease^23^.

A direct association between folate status and insulin resistance has not been well established, although it is known that a low folate status is associated with an increased risk of neural tube defects, cancer, and coronary heart disease^9^. Some recent evidence suggested that folate deficiency of parents may predispose offspring to insulin resistance and diabetes. An animal study using an inbred C57BL/6 mouse model reported that low paternal dietary folate altered levels of DNA methylation in spermatogenesis levels with consequences for offspring health, such as insulin resistance, and diabetes. They found that low-folate diet could affect the methylation status of *Nkx2-2, Uts2, Cyp2e1*, which are crucial genes associated with diabetes^24^. Kevin *et al*. reported that male offspring of sheep fed folate deficient diet exhibited clear signs of insulin resistance that were independent of differences in adiposity, but not female offspring. They proposed that the phenotypic sex differences might be explained by the fact that over half of the affected loci were specific to males^25^. There have been similar findings in a subsequent human study. The human study using data from the Boston Birth Cohort indicated that low maternal serum folate concentrations were associated with an increase in the concentrations of insulin (a marker of insulin resistance) and a reduction in the adiponectin to leptin ratio (a marker of insulin sensitivity) in offspring^13^. However, in one Indian cohort study, Yajnik *et al*. reported higher maternal erythrocyte folate concentration was positively associated with HOMA-IR in the offspring^26^.

Besides these data pertaining maternal folate status and insulin resistance in offsprings, evidence from human studies, particularly studies on large population with normal weight and without metabolic syndrome, are very limited, with only a few studies on subjects with metabolic syndrome or overweight/obesity. Emanuela *et al*. reported folate (5 mg/d) plus vitamin B12 (500 ug/d) supplementation for 1 month among patients with metabolic syndrome decreased homocysteine levels by 27.8%, insulin levels by (25.6%), and HOMA-IR levels by 27%^16^. Similar results were observed in an unmasked randomized placebo-controlled trial. The study showed that 3 months of supplementation with folic acid (2.5 mg) significantly reduced plasma insulin concentrations and HOMA-IR index in overweight healthy participants^17^. Another clinical trial with small sample size (n = 60) suggested that folate supplementation (5 mg/d) for 6 months among women with cervical intraepithelial neoplasia resulted in disease regression and decreased plasma homocysteine and serum insulin^18^.

In this study, we examined relationships between serum folate and insulin resistance and sensitivity biomarkers among U.S. nondiabetic adults. We observed consistently associations of serum folate with those biomarkers: significant inverse relationship with HOMA-IR and insulin and a positive association of with G/I ratio. These associations remain significant after adjusting for groups of potential confounding factors as described in the three models. These associations observed between serum folate and insulin resistance in the overall population persistently existed after stratifying by gender, race/ethnicity, and drinking. Considering homocysteine may promote insulin resistance^27–29^ and long-term ingestion of large quantities of alcohol causes inhibition of methionine synthase activity^30^, we tested whether alcohol consumption moderates the associations between serum folate and insulin resistance. We found that drinking did not moderate the association between serum folate and insulin resistance, which suggested that methionine synthase was not involved in the association between serum folate and insulin resistance.

These findings have potentially important clinical and public health implications. Insulin resistance is a major risk factor for development of type 2 diabetes, coronary heart disease, stroke, breast cancer, and kidney disease^31^. Identifying risk factors for insulin resistance is important for early prevention and intervention. Our results show an inverse relationship between serum folate and insulin resistance in non-diabetic patients. If a causal link between serum folate and insulin resistance is confirmed, folic acid could be utilized not only for the well-known prevention of neural tube defects, but also for the improvement of insulin resistance.

There are several limitations existing in our findings. First, NHANES is a cross-sectional study, and thus it does not allow inferences regarding the temporality of events and the causality between serum folate and insulin resistance. In other words, this study design cannot determine whether serum folate reduces insulin resistance or whether insulin resistance could cause reduces serum folate uptake. Second, some residual confounding may not be ruled out as a potential explanation of our findings. Lastly, the measurements, HOMA-IR, insulin, and GI ratio were conducted in fasting state, whereas euglycemic-hyperinsulinemic clamp tests insulin resistance in an insulin-stimulated state, but the data was not available in NHANES. Despite these limitations, our study has several strengths: NHANES employs a rigorous sampling design, an extensive quality assurance and quality control procedures, and a representative general population of United States; our study represents a large sample size and population-based study; samples in HNANES were collected in the morning session, which is likely to reduce differences because of diurnal variation. We examined those associations with three different models which controlled for different potential covariates, and those associations remain consistently across those models, indicating genuine associations between serum folate and insulin resistance.

In conclusion, this cross-sectional analysis of a representative sample of U.S. nondiabetic adults showed that serum folate was inversely associated with an increased prevalence of insulin resistance, indicating potential benefits for the prevention of insulin resistance by improving serum folate status.
